# Vagus Nerve through **α**7 nAChR Modulates Lung Infection and Inflammation: Models, Cells, and Signals

**DOI:** 10.1155/2014/283525

**Published:** 2014-07-20

**Authors:** Haiya Wu, Ling Li, Xiao Su

**Affiliations:** Unit of Respiratory Infection and Immunity, Institut Pasteur of Shanghai, Chinese Academy of Sciences, B104, Life Science Research Building, 320 Yueyang Road, Shanghai 200031, China

## Abstract

Cholinergic anti-inflammatory pathway (CAP) bridges immune and nervous systems and plays pleiotropic roles in modulating inflammation in animal models by targeting different immune, proinflammatory, epithelial, endothelial, stem, and progenitor cells and signaling pathways. Acute lung injury (ALI) is a devastating inflammatory disease. It is pathogenically heterogeneous and involves many cells and signaling pathways. Here, we emphasized the research regarding the modulatory effects of CAP on animal models, cell population, and signaling pathways that involved in the pathogenesis of ALI. By comparing the differential effects of CAP on systemic and pulmonary inflammation, we postulated that a pulmonary parasympathetic inflammatory reflex is formed to sense and respond to pathogens in the lung. Work targeting the formation and function of pulmonary parasympathetic inflammatory reflex would extend our understanding of how vagus nerve senses, recognizes, and fights with pathogens and inflammatory responses.

## 1. Introduction

From 2000, Tracey and colleagues have been working on the mechanisms by which electric stimulation of vagus nerve suppresses activation of NF-*κ*B and production of proinflammatory cytokines in *α*7 nicotinic acetylcholine receptor- (*α*7 nAChR-) expressing macrophages and lessens severity of sepsis in animal models [1, 2]. These findings led to establishment of a novel theory of cholinergic anti-inflammatory pathway (CAP) [1]. Most experiments regarding the modulatory effects of CAP on inflammation were tested in the models of sepsis, a syndrome of systemic proinflammatory responses. The experimental results support that spleen is the functional hub of CAP. In 2011, Tracey and colleagues found that innervation of vagus nerve in the spleen, CHAT-expressing T lymphocytes, and *α*7 nAChR-expressing macrophages forms a neural circuit to finely tune the proinflammatory responses [3]. In this review, we summarized the progress regarding the modulatory effects of CAP on inflammation and pointed out the future research directions towards brain center of CAP, activation of *β*2 adrenergic receptor, synthesis of acetylcholine in the T lymphocytes, and others.

ALI is a devastating inflammatory disease [4]. It is pathogenically heterogeneous and involves many cells and signaling pathways. From 2007, the modulatory effects of CAP have been tested in a variety of animal models with ALI [5]. Activation of CAP also affects many types of cells and signaling pathways involved in ALI. In this review, we compared the deferential pulmonary inflammatory responses during sepsis (systemic) and ALI (local). Considering different features of modulatory effects of CAP on pulmonary inflammatory responses, we put forward a new working model, pulmonary parasympathetic inflammatory reflex, to extrapolate how vagus nerve through *α*7 nAChR modulates acute lung infection, inflammation, and injury. The pulmonary parasympathetic inflammatory reflex works locally and may not require spleen. In accordance with this assumption, vagus nerve coupling with *α*7 nAChR-expressing resident macrophages also modulates intestinal inflammation independent of spleen [6]. Thus, future studying local modulatory effects of CAP on inflammation may be an emerging avenue to explore how vagus nerve senses, recognizes, and responds to pathogens.

## 2. Cholinergic Anti-Inflammatory Pathway

### 2.1. Inflammatory Reflex and CAP

Studies suggest that afferent and efferent vagus nerves, *α*7 nAChR-expressing inflammatory cells, and central vagal nucleus in the brain form an inflammatory reflex that could finely tune inflammation and immunity [2, 7]. Strictly speaking, CAP is the efferent arm of vagal inflammatory reflex and spleen may be the anti-inflammatory hub in this neural circuit [8–10]. Activation of this pathway provides the host with a fast, discrete, and localized means of modulating the inflammatory and immune responses in variety of animal models [11–13].

### 2.2. The Role of Spleen in Inflammatory Reflex

**Figure 1:**
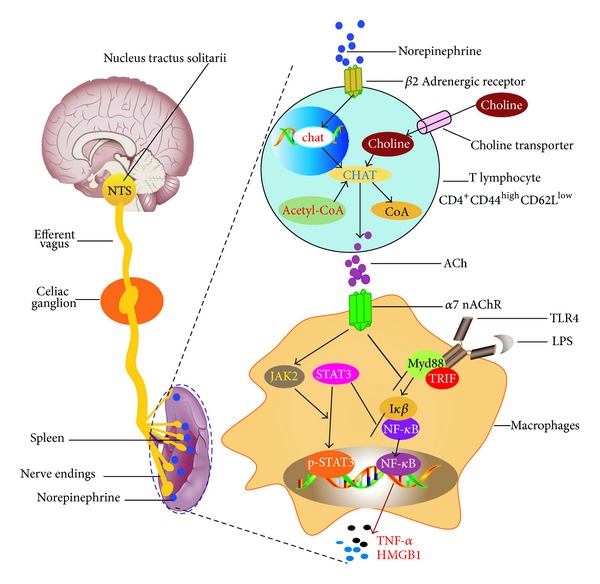
The hypothetical model of cholinergic anti-inflammatory pathway.

As Figure 1 shows, in the vagal inflammatory reflex, the sensory neurons may sense the changes of pathogen associated molecular patterns (PAMPs) or damage associated molecule patterns (DAMPs) in the peripheral afferent vagal nerve endings and then feedback to nucleus tractus solitarii (NTS) in the brain stem. After the information is processed in the NTS, the efferent vagus nerve transmits integrated information by action potentials to the celiac ganglion and then delivers in the spleen. Anatomically, the splenic vagus nerve endings are closely in contact with a group of *β*2 adrenergic receptor- (*β*2 AR-) expressing T memory lymphocytes (CD4^+^CD44^high^CD62L^low^) and release norepinephrine (NE), a sympathetic neurotransmitter. NE activates *β*2 AR in the T lymphocytes, initiates transcription of choline acetyltransferase (ChAT), and synthesizes acetylcholine (ACh). ACh could activate splenic *α*7 nAChR-expressing macrophages, inhibit NF-*κ*B activity, promote STAT3 phosphorylation [14], and therefore dampen proinflammatory cytokine production (especially TNF-*α* and HMGB1) [2, 3, 12, 15, 16].

We have to point out that Figure 1 is a hypothetical model that shows a direct connection of the efferent vagus nerve to the spleen via the celiac ganglion [3]. However, a recent finding has demonstrated there was no neural connection from the vagus to splenic sympathetic by neuroanatomical tract tracing and neurophysiological measurements [17]. Moreover, one study has showed that sympathetic nerves rather than vagi contribute to anti-inflammatory effects revealed by a LPS-challenged splanchnic-nerve or vagus nerve-cut rat model to compare changes of splanchnic sympathetic nerve activity and peripheral blood TNF-*α* [18, 19]. But this study has its limitations, for example, short experimentation (1-2 h), small sample size, and unknown mechanisms. More recently, Torres-Rosas et al. have found that electroacupuncture controls systemic inflammation in sepsis via the sciatic and vagus nerves and catecholamines from the adrenal glands [20]. Therefore, the controversy regarding anti-inflammatory roles of vagus and sympathetic nerves should be sorted out in the future study.

### 2.3. Information-Integrating Center of Inflammatory Reflex

It should be mentioned that vagus nerve originates from medullar oblongata, which consists of four nuclei: dorsal nucleus, nucleus ambiguous, NTS, and spinal nucleus of trigeminal nerve [21, 22]. About 80% afferent sensory fibers are contained in the vagus nerve and responsible for transmission of the information to the NTS [21]. For example, animals were intravenously or intraperitoneally challenged with LPS or IL-1*β* could induce c-fos expression in the nodose ganglia and the NTS [23, 24]. NTS also plays a very important role in projecting information to the nuclei (including the locus ceruleus and dorsal raphe nuclei) of the brain [21]. It is unknown whether and how PAMPs or DAMPS can be recognized by the afferent vagus nerve endings in the lung and how NTS processes the information that is collected from the lung during infection and inflammation.

### 2.4. Transcription of ChAT in *β*2 AR-Expressing T Lymphocytes

Upon the vagus nerve stimulation, transcription of ChAT gene in the splenic *β*2 AR-expressing T lymphocytes might be regulated by cAMP, which is a major second messenger following activation of *β*2 AR [25, 26]. However, study has also shown that stimulation of efferent vagus nerve induces plasma norepinephrine via the *α*7 nAChR in a mouse model [27]. This finding raises a possibility that transcription of ChAT and biosynthesis of ACh in the splenic lymphocytes are positively regulated by *β*2 AR or *α*7 nAChR. Experimental data have demonstrated that *β*2 AR-expressing CD3 lymphocytes and *α*7 nAChR-expressing CD11b/c monocyte or macrophages are present in the spleen. The defects in response to *β*2 AR or *α*7 nAChR stimulation or quantity in the spleen lead to the dysfunction of inflammation resolution and postoperative cognition decline [28]. In addition, the *β*2 agonist is well recognized for its anti-inflammatory property for ALI [29, 30]. Whether this protective effect of *β*2 agonist on ALI [31] is via activation of splenic *β*2 AR-expressing T lymphocytes or CAP requires to be investigated.

### 2.5. Synthesis of Acetylcholine in Nonneuronal Cells

It needs to emphasize the important role of high-affinity choline transporter (CHT1) or choline transporter like proteins (CTLs) in the process of ACh synthesis in the nonneuronal cells (e.g., lymphocytes and lung cells) [32–34]. ACh is synthesized from choline and acetyl-CoA by the enzyme choline acetyltransferase (ChAT), and this event may be limited by choline availability [35]. In neurons, loss of CHT-mediated presynaptic choline uptake might result in neonatal lethality [36]. ChAT contains nuclear localization signals and is also localized in the nuclei of neural and nonneuronal cells [37]. Enzymatic activity and nuclear translocation of ChAT are required for its transcriptional enhancement of CHT gene [37]. Pulmonary nonneuronal cholinergic system (including ChAT-, CTLs-, VAChT-, and OCT-mRNA) is downregulated in acute allergic airway inflammation [35], suggesting that synthesis of ACh is regulated locally during inflammation.

## 3. Acute Lung Inflammation and Injury and Modulatory Effects of CAP

### 3.1. Acute Lung Inflammation and Injury

Adult respiratory distress syndrome (ARDS), characterized by ALI, has a mortality of 40% even if the patients receive advanced intensive care [4]. Pneumonia, severe sepsis, and acid aspiration are the most serious causes of ARDS [4, 38, 39]. Gram-negative sepsis derived ALI is characterized by neutrophil alveolitis and increased permeability of the lung microvascular endothelial and alveolar epithelial barriers [40–42]. Aspiration of gastric contents is reported to be associated with a 26–36% incidence of ARDS [43, 44]. Aspirated hydrochloric acid may evoke direct damage to the alveolar-capillary membrane and promote adhesion, activation, and sequestration of neutrophils.

### 3.2. Direct and Indirect Animal Models of Acute Lung Inflammation and Injury

**Figure 2:**
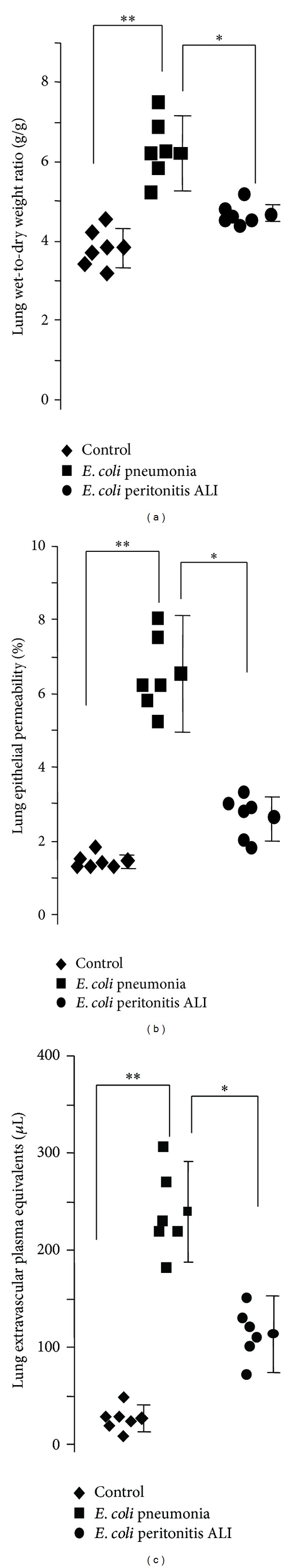
Different challenge routes of pathogen affect the outcome of acute lung inflammation. Data were pooled from 6 mice in each group. Values are presented as mean ± SD. One-way analysis of variance (ANOVA) with post hoc Bonferroni test was used for statistical analysis (level set at *P* < 0.05). The committee on Animal Research of Institut Pasteur of Shanghai, Chinese Academy of Sciences approved all the protocol.

Alveolar epithelial cells are the main target cells in the epithelial respiratory compartment exposed to noxious substances such as* E. coli* or acid [45]. Injury to the alveolar epithelial barrier is a major determinant of severity of clinical ALI [46, 47]. Our experiments have demonstrated that, at the same dosage, intratracheal challenge of* E. coli* could induce much severe lung inflammation than intraperitoneal challenge of* E. coli*. As Figure 2 shows, mice were divided into three groups: control group received PBS;* E. coli* pneumonia group received an intratracheal challenge of* E. coli* (10^7^ cfu);* E. coli* peritonitis ALI group was given an intraperitoneal challenge of* E. coli *(10^7^ cfu). All mice were also given I^125^-albumin intratracheally or intravenously to measure lung wet-to-dry weight ratio and lung epithelial and endothelial permeability as previously reported [5, 48]. At 4 h after challenge, three parameters were markedly higher in the* E. coli* pneumonia compared to* E. coli* peritonitis ALI.

### 3.3. Category of ALI

ALI experimental models can be categorized into direct and indirect lung injury based on the route of insults. Acid-induced ALI, LPS-induced ALI,* E. coli* pneumonia, and other experimental ALI models were considered direct models because the injurious agents (such as HCl, bleomycin, endotoxin,* E. coli*, and influenza virus) were instilled into the air spaces with initial direct contact with pulmonary epithelium [5, 49–51]. Ventilator-induced ALI caused by overstretch of lung epithelial cells is also considered as direct lung injury [52–54]. Thiourea-induced lung vascular injury [55], oleic acid-induced ALI [56, 57], peritonitis-induced ALI (including cecal ligation and puncture (CLP)) [16], and transfusion-related ALI (TRALI) (by intravenous MHC I monoclonal antibody) [58] were considered as indirect models because the injurious agents initially interacted with the lung endothelium after intravenous challenge [55].

### 3.4. Different Effects of CAP on Lung Cytokines: Intratracheal versus Intravenous Insult

The modulatory effects of CAP on proinflammatory cytokines also alter when the challenge route of pathogens is different. Numerous studies have demonstrated that TNF-*α* is a proinflammatory cytokine and is well regulated by CAP. The spleen is identified as the source of 90% of the serum TNF during endotoxemia and in particular the marginal zone- and red pulp-macrophages of the spleen [10, 59]. Compartmentalization of TNF-*α* in the blood or alveolus is dependent on route of LPS challenge. For example, intravenous endotoxin significantly increases TNF-*α* production in the spleen by a factor of 30 as compared with six- and twofold increases in the lung and liver, respectively. Vagus nerve stimulation significantly reduces TNF levels in the spleen (94%) and liver (40%) but not in the lung (20%). However, in a lung injury model by an intratracheal challenge, compartmentalization of TNF-*α* in alveolus is preserved before alveolar-capillary injury [60]. Once compartmentalization of alveolar TNF-*α* is lost, injured lung may contribute to a systemic inflammatory response and subsequent multiorgan failure [60]. Similarly, intratracheal LPS induced a significant increase in MIP-2 in BAL fluid, whereas MIP-2 in the plasma was not detectable. In contrast, intravenous LPS caused a marked increase in plasma MIP-2, whereas only a small elevation of MIP-2 concentration in BAL fluid was observed [61]. In a LPS-induced ALI (intratracheal), administration of *α*7 nAChR agonists could inhibit NF-*κ*B activity in the BAL proinflammatory cells and reduce both TNF-*α* and MIP-2 levels in the BAL [5]. Vagotomy and deficiency of *α*7 nAChR worsen lung inflammation [5, 50].

### 3.5. The Modulatory Effects of CAP on Animal Models of ALI Are Dependent on PRR (Table 1)

By analyzing Table 1, we can conclude that activation of CAP might affect the development of lung infection, inflammation, or injury in a PRR- (pattern recognition receptors-) dependent manner. For example, nicotine administration worsens Gram-positive bacterial pneumonia (TLR2) [50] and influenza viral pneumonia (TLR3, TLR7, or RIG-I-MAVS) [62–64] but improves Gram-negative bacterial pneumonia or LPS-induced ALI (TLR4) [65]. It has to be noted that activation of *α*7 nAChR universally suppresses TLR2, TLR3, TLR4, or TLR9 agonist (rather than live pathogens) induced TNF-*α* production in monocytes [66]. These findings suggest that vagus nerve through *α*7 nAChR responds to PAMPs or DAMPs differently. Therefore, vagus nerve may play pleiotropic roles in modulating lung infection and inflammation.

### 3.6. Opposite Effects of CAP on Lung Infection and Inflammation

The discovery that splenectomy inactivates CAP strongly supports that spleen determines the function of CAP [9]. In the splenectomized animals, nicotine therapy worsens animals with lethal polymicrobial sepsis [9]. This finding suggests that once CAP is dysfunctional, activation of *α*7 nAChR would paradoxically compromise immunity and worsen lung infection. Traumatic brain injury or stroke might cause functional impairment of CAP and activation of *α*7 nAChR worsened Gram-negative bacterial pneumonia [59, 67].

## 4. Pulmonary Parasympathetic Inflammatory Reflex

### 4.1. Vagus Nerve Helps Sensing, Recognizing, and Responding to Pathogens

We have to mention that classical CAP theory was mostly tested in the experimental models of sepsis (intravenous LPS) or CLP peritonitis animal models in which spleen is required for dampening inflammation; however, these models only present mild lung inflammation which is manifested by less impressive intra-alveolar inflammation and hyaline membrane formation [68]. Different from the regulatory effects of the classical CAP on sepsis, vagus nerve might modulate lung infection and inflammation using new machinery:* pulmonary parasympathetic inflammatory reflex* [69], and the spleen may not be involved in this regulatory mechanism.

**Figure 3:**
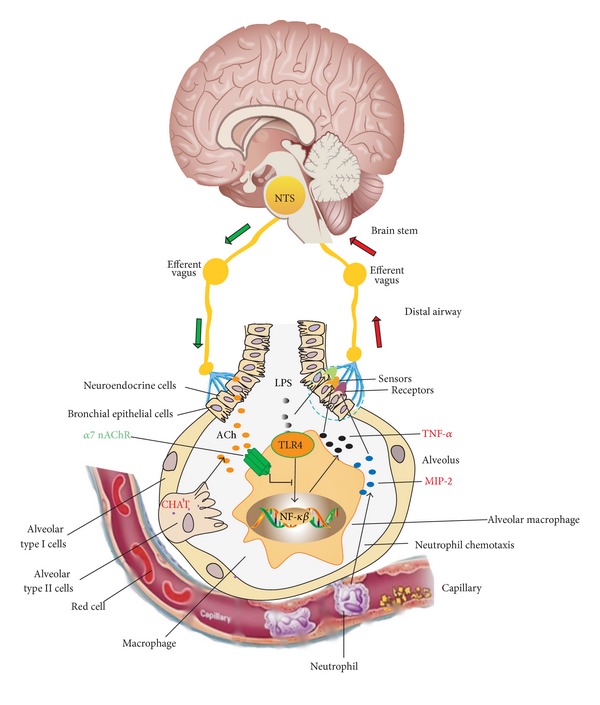
The working model of pulmonary parasympathetic inflammatory reflex.

As illustrated in Figure 3, the pulmonary parasympathetic inflammatory reflex may consist of three components: the afferent arc residing in the distal airway or alveolus; the NTS information-integrating center in the brain stem; and the efferent arc innervating the distal lung epithelial cells. Vagus nerve endings are reported to innervate the distal airway of the lung, possibly in the alveoli [70, 71] (though it is entirely unclear how efferent fibers traveling in the vagus nerve might exert influence upon the alveolar region), where varieties of sensors or PRR in the vagal afferent arc are located. Via this apparatus, mechanical, chemical, biological, and other stimuli in the alveoli can be sensed. Sensory neurons express TLR3, 4, 7, and 9, which can recognize different pathogens [72–74]. Lung neuroendocrine cells also are complex airway sensors, which are predominantly innervated by vagal afferent fibers derived from the nodose ganglion [75]. The information is transmitted via the afferent arm to NTS, a processing center, which is capable of differentiating types of infection, inflammation, or challenges. After processing, the active potentials are remitted from NTS to the alveoli via the vagal efferent arc. The vagal nerve endings could synthesize and release ACh, which in turn activates *α*7 nAChR in the proinflammatory cells (e.g., macrophages and neutrophils) or epithelial cells to regulate the production of proinflammatory cytokines via NF-*κ*B or other signaling pathways.

### 4.2. Recruitment of *α*7 nAChR-Expressing Cells and Nonneural ACh in the Lung

During lung infection and inflammation, alveolar macrophages produce MIP-2, a key chemokine, which could attract neutrophil migrating into the alveoli [76]. These infiltrated neutrophils also express *α*7 nAChR. Apart from release of ACh from the vagal nerve endings in the distal airway, lung epithelial cells, immune cells, and neuroendocrine cells also produce nonneuronal ACh [32, 35, 77]. *α*7 nAChR in bronchial epithelial cells can be upregulated by the stimulation of nicotinic agonists [78, 79]. This positive feedback between acetylcholine and *α*7 nAChR might facilitates maintenance of concentration of acetylcholine in the alveoli.

### 4.3. The Role of Local CAP Is Emerging

In addition, the theory of classical CAP has also been challenged by recent researches. In a rat model, one group reported that vagal efferent neurons in the rat neither synapse with splenic sympathetic neurons nor drive their ongoing activity using vagal terminals anterograde and Fast Blue labeling technology and electrophysiological stimulation [17]. A recent study has shown that gastrointestinal CAP plays a protective role in a mouse of postoperative ileus [6]. In this study, denervation of spleen and depletion of T lymphocytes could not deactivate the protective property of vagus nerve stimulation. Anterograde labeling revealed that vagal efferents closely make contacts between cholinergic myenteric neurons and resident *α*7 nAChR-expressing macrophages. Therefore, the protective effects are attributable to local vagal nerve innervation and resident macrophages independent of spleen [6].

## 5. Modulatory Effects of CAP on Cells Involved in Lung Inflammation

### 5.1. Cells Involved in Acute Lung Inflammation

Macrophages (monocytes), neutrophils, mononuclear cells, epithelial cells, endothelial cells, hematopoietic stem cells, mesenchymal stem cells, endothelial progenitor cells, T lymphocytes, and fibroblasts play roles in the different phases of lung inflammation and repair [4, 5, 49, 68, 76, 80, 81].

### 5.2. Modulatory Effects of CAP on Different Cells

Activation of *α*7 nAChR could modulate inflammatory responses in variety of types of cells and affect the development of inflammatory models (Table 2).

### 5.3. Modulatory Effects of Activation of *α*7 nAChR on Inflammatory Cells May Be Dynamic

On average, 20–25% of total cells are *α*7 nAChR-expressing cells in the bone marrow (BM), blood, spleen, lymph nodes, and Peyer's patches [82]. Lung *α*7 nAChR^+^Gr1 or *α*7 nAChR^+^CD11b^+^ granulocytes (neutrophils and monocytes) were increased to 40% after being infected with* E. coli* [50], suggesting that more granulocytes migrate into the lung. In addition, vagus nerve stimulation significantly attenuates CD11b^+^ cells in the spleen during sepsis [83]. These findings support that *α*7 nAChR-expressing proinflammatory cells can dynamically migrate among lung, spleen, and other organs during different stages of inflammation [84]. This dynamic movement of *α*7 nAChR-expressing cells might facilitate them being activated by acetylcholine released from the vagus nerve.

## 6. Modulatory Effects of CAP on Signaling Pathways

### 6.1. Signaling Pathways Involved in Acute Lung Inflammation

It was reported that activation of p38 MAPK, AKT1, and NF-*κ*B in neutrophils contributes to ALI [85]. Lack of AKT1 could worsen acid, LPS, or bacteria induced acute lung infection and inflammation [86–88]. LPS activates the STAT kinases, Src, and JAK. LPS treatment could activate STAT3 in the resident lung cells and recruited inflammatory cells [89]. In a rat model of intrapulmonary deposition of IgG immune complexes, STAT3 activation was dramatically suppressed by depletion of neutrophils or lung macrophages, resulting in reduced gene expression of IL-6 and IL-10 in whole lung tissues [90].

### 6.2. Modulatory Effects of Activation of *α*7 nAChR on Signaling Pathways

Activation of *α*7 nAChR in macrophages, monocytes, and other immune cells may downregulate production of proinflammatory cytokines and attenuate the inflammatory responses by several possible mechanisms: NF-*κ*B activation, JAK-STAT3 pathway, and PI3K-AKT1 pathway. Activation of *α*7 nAChR by its agonists in monocytes and macrophages could reduce nuclear translocation of NF-*κ*B and the transcription of proinflammatory cytokines [1, 2, 91, 92]. In the sepsis and lung injury mouse models, administration of *α*7 nAChR agonist also suppresses activation of NF-*κ*B [5, 16]; however, one study showed that vagus nerve electrical stimulus could attenuate the proinflammatory cytokine responses* in vivo* but did not decrease the NF-*κ*B activation [93]. Whether activation of *α*7 nAChR affects TLR4 signaling pathway, for example, MyD88, TRIF, IRAK, TARF, and other adaptor proteins in the proinflammatory cells, needs further study. The modulatory effects of activation of *α*7 nAChR on signaling pathways are summarized in Table 3.

### 6.3. Spatial and Temporal Effects of *α*7 nAChR Activation on p-STAT3

Anti-inflammatory effect of nicotine in murine macrophages acts through the recruitment of JAK2 to the *α*7 nAChR and subsequent phosphorylation of JAK2, thereby initiating the anti-inflammatory STAT3 cascade [14]. JAK2 inhibitor AG490 inhibited the anti-inflammatory effect of GTS-21 in the human PBMCs (peripheral blood mononuclear cells) [94], suggesting that p-STAT3 mediates inhibitory role of activation of *α*7 nAChR. However, in an endothelial cell line, GTS-21 significantly reduced STAT3 activation by phosphorylation and DNA binding [95]. In the splenocytes or myocardium tissue, cardiac troponin I (TnI) induced STAT3 activation and IL-6 is inhibited by nicotine [96]. In macrophage cell line, both *α*7 nAChR activation and inhibition of JAK2 blunt STAT3 phosphorylation. Inhibition of STAT3 phosphorylation mimicked the *α*7 nAChR signaling, inhibiting NF-*κ*B and cytokine production in macrophages. These findings suggest the proinflammatory role of p-STAT3. In addition, unphosphorylated STAT3 might compete with NF-*κ*B. Inhibition of STAT3 protein expression enhanced cytokine production and abrogated *α*7 nAChR signaling [97].

### 6.4. Modulatory Effects of *α*7 nAChR Activation Might Involve CREB and c-fos

It has been assumed that interaction between *α*7 nAChR and adenylate cyclase 6 increases intracellular cAMP, a secondary messenger, which in turn promotes phosphorylation of CREB. P-CREB translocates into the nucleus and initiates transcription of* c-fos*, an early response gene. Activation of c-fos could inhibit NF-*κ*B activity and production of proinflammatory cytokines [11, 59]. So far, there is no scientific evidence to prove this hypothesis, but some previous findings indicate that it might be testable. For example, in epithelial cells, *α*7 nAChR physically binds adenylate cyclase 6 [98]. In response to LPS stimulation, Fos^−/−^ macrophages and mice showed significantly enhanced production of TNF-*α*, IL-6, and IL-12 p40 but reduced production of the anti-inflammatory cytokine IL-10 compared with wildtype controls. Activation of c-fos inhibits NF-*κ*B activity [99].

## 7. Concluding Remarks

How nervous system, especially vagus nerve, modulates inflammation and immunity has been a puzzle for many years. In past decade, a large body of evidence has shown that the classical CAP could systemically modulate proinflammatory responses via spleen. More recently, the regulatory role of local CAP is emerging and challenging. The immediate questions we have to answer are the following. How vagus nerve senses the PAMPs or DAMPs in the airspaces of the lung? What sensors and receptors are used by sensory nerve endings during this process? To where and how does vagus nerve send the pathogenic signals? How are pathogenic signals being integrated or transformed in the brain center? What are targeting cell population of vagus nerve in the deferent stages of infection and inflammation? How signaling pathways are finely tuned by vagus nerve spatially and temporally during infection and inflammation? In summary, the overall task of this review is to extend our understanding of how nervous and immune systems work collaboratively during infection and inflammation.

## Figures and Tables

**Table 1:** Modulatory effects of CAP on animal models of ALI.

Animal models	Inducer	Route	Injury type	Major effects of CAP	Outcome	References
Acid-induced ALI (mouse and rats) [49]	HCl acid	IT	Direct: lung epithelial cells	(i) Activation of *α*7 nAChR by nicotine, choline, and PNU-282987 (a specific *α*7 nAChR agonist) decreased excess lung water and lung vascular permeability and reduced protein concentration in the BAL. (ii) Deficiency of *α*7 nAChR resulted in a 2-fold increase in excess lung water and lung vascular permeability.	Protective	[5]

LPS-induced ALI (mouse)	LPS	IT	Direct: lung epithelial cells	(i) Nicotine treatment reduced the LPS-mediated infiltration of leukocytes and edema as evidenced by decreased BALF inflammatory cells, myeloperoxidase, and protein. (ii) Nicotine also downregulated lung production of proinflammatory chemokines and cytokines. (iii) Intranasal inoculation with GTS-21 also dose dependently inhibited TNF-alpha release into the lung compartment after intrapulmonary delivery of LPS in mice *in vivo*.	Protective	[50, 100–102]

*Gram-negative * *E. coli* pneumonia (mouse)	*E. coli *	IT	Direct: lung epithelial cells	(i) Administration of *α*7 nAChR agonists reduced bronchoalveolar lavage MIP-2 production and transalveolar neutrophil migration and reduced mortality in *E. coli* pneumonia. (ii) Vagal denervation increased MIP-2 production and airway neutrophil accumulation and increased mortality. (iii) *α*7 nAChR deficient mice developed severe lung injury and had higher mortality compared with wildtype mice.	Protective	[50]

*Gram-negative* *P. aeruginosa pneumonia* (mouse)	*P. aeruginosa (PA) *	Stroke, then IT PA	Direct: lung epithelial cells	(i) Exacerbation of *P. aeruginosa*-induced lung injury and mortality by prior stroke is reduced by loss of *α*7 nAChR receptors. (ii) Genetic deletion of the *α*7 nAChR attenuates the effect of stroke on bacterial clearance in *P. aeruginosa* pneumonia. (iii) Pretreatment with PNU-282987, a pharmacologic activator of the *α*7 nAChR significantly increased lung injury caused by *P. aeruginosa* pneumonia, significantly decreased the release of KC, a major neutrophil chemokine, and significantly decreased intracellular bacterial killing by a mouse alveolar macrophage cell line and primary mouse neutrophils.	Worse	[67]

Gram-positive bacterial pneumonia (mouse)	*Streptococcus pneumoniae *	IT	Direct: lung epithelial cells	(i) Nicotine treatment was associated with a transiently enhanced growth of *S. pneumoniae* in both lungs and blood. (ii) Mice treated with nicotine showed enhanced lung inflammation at 24 h after infection. (iii) Both lung and plasma concentrations of the proinflammatory cytokines tumor necrosis factor-alpha and interferon-gamma were higher in nicotine-treated animals at this time point.	Worse	[65]

Peritonitis-induced acute lung (rats)	Feces	CLP	Indirect: lung endothelial cells	(i) Posttreatment by VNS increased survival peritonitis-induced ALI. (ii) Nicotine administration increased lung PMN infiltration and mortality. (iii) Nicotine induced bacterial clearance impairment.	VNS: protective Nicotine: worse	[103]

Ventilator-induced lung injury (VILI) (mouse and rats)	Shear forces	MV	Direct: lung epithelial cells	(i) Pharmacological pretreatment with PNU-282987 strongly decreased lung injury and lung IL-6 and substance P contents and nearly abolished the increase in plasmatic IL-6 levels. (ii) Vagal stimulation was able to maintain the respiratory parameters close to those obtained in controls and reduced lung inflammation except when associated to nicotinic receptor blockader MLA. (iii) Stimulation of the cholinergic anti-inflammatory pathway with GTS-21 attenuates MV-induced release of TNF-*α*, which was associated with reduced lung injury. (iv) Vagotomy exacerbates lung injury from VILI in mice as demonstrated by increased wet-to-dry ratio, infiltration of neutrophils, and increased IL-6.	Protective	[52–54]

Sepsis + VILI (rats)	LPS + shear forces	2-hit LPS IV MV	Direct: lung epithelial cells	(i) Vagotomy enhanced the LPS-induced pulmonary, but not systemic proinflammatory cytokine SP rats, but not in MV animals (TNF-*α*, IL-6, and KC compared to sham), and resulted in decreased pO_2_ (compared to sham-operated animals). (ii) VNS did not affect any of the studied parameters in both SP and MV animals. (iii) MV with moderate tidal volumes potentiates the pulmonary inflammatory response elicited by systemic LPS administration. (iv) No beneficial effects of vagus nerve stimulation performed following LPS administration were found.	VNS is protective in LPS challenge but not in LPS + MV model	[104]

Oleic-acid induced ALI (dogs) [49, 57]	Oleic acid	IV	Indirect: lung endothelial cells	(i) In the dogs with normal lungs, bilateral vagotomy per se did not cause lung injury during 3 h of observation. (ii) In oleic acid-induced ALI, vagotomy significantly deteriorated pulmonary edema by increasing pulmonary intravascular pressures. (iii) Inhibition of vagal or sympathetic innervation will aggravate pulmonary edema in the dog.	Protective	[105]

Influenza virus-induced ALI	Influenza virus	IN	Direct	(i) The airway reactivity to acetylcholine at 2 weeks after infection was increased by 2.3 to 6.5 times the normal value in terms of the acetylcholine provocative concentration after influenza viral infection. (ii) The virus was apparently transported from the respiratory mucosa to the CNS directly and decussately via the vagus nerve and centrifugally to the vagal ganglion of the vagotomized side. (iii) Nicotine suppressed the migration of leukocytes to the inflammation/infection site and increased the influenza titer in the lung.	Worse	[62–64]

LPS: lipopolysaccharide; IT: intratracheally; IN: intranasally; IV: intravenously; CLP: cecal ligation puncture; MV: mechanical ventilation; VILI: ventilator-induced lung injury; TNF-*α*: tumor necrosis factor; IL-6: interleukin-6; KC: keratinocyte chemoattractant; MIP-2: monocyte inflammatory protein-2; VNS: vagus nerve stimulation; BAL: bronchoalveolar lavage; MLA: methyllycaconitine; SP: spontaneously breathing.

**Table 2:** Modulatory effects of CAP on different cell populations.

Cells	Species	Models	Interventions	Major effects	Outcome	References
Macrophages	Human Mouse	Sepsis; CLP; acid-induced ALI; pneumonia	*α*7 nAChR agonists, VNS, and genetic depletion	(i) *α*7 nAChR is expressed on the surface of human and mouse alveolar macrophages. (ii) Cholinergic agonists inhibit TNF-*α* production and HMGB1 release from macrophages. (iii) Vagus nerve stimulation does not inhibit TNF production in *α*7-subunit-deficient mice. (iv) Increased cytokine production in *α*7-subunit-deficient mice during endotoxaemia. (v) Nicotinic treatment prevents lethal endotoxemia. (vi) Deficiency of *α*7 nAChR increases BAL proinflammatory cytokines.	Protective effects of CAP on sepsis and ALI	[2, 5, 16, 50]

Monocytes	Human	*In vitro* cell culture	*α*7 nAChR agonists	(i) GTS-21 attenuated TNF production in monocytes stimulated with peptidoglycan, polyinosinic-polycytidylic acid, CpG, HMGB1, and RAGE-modified albumin. (ii) GTS-21 decreased TNF levels in endotoxin-stimulated whole blood obtained from patients with severe sepsis. (iii) Nicotine inhibited the actions of AGE-2 and AGE-3. Nonselective and selective *α*7 nAChR antagonists, mecamylamine and *α*-bungarotoxin, reversed the inhibitory effects of nicotine, suggesting the involvement of *α*7 nAChR stimulation.	Protective effects of CAP on sepsis and ALI	[66, 106]

Neutrophils	Mouse	Sepsis; LPS-induced ALI; acid-induced ALI	*α*7 nAChR agonists, VNS, and genetic depletion	(i) *α*7 nAChR is expressed on the surface of neutrophils. (ii) Administration of nicotine, a pharmacologic agonist of the cholinergic anti-inflammatory pathway, significantly reduces levels of CD11b, a *β*2-integrin involved in cell adhesion and leukocyte chemotaxis, on the surface of neutrophils in a dose-dependent manner and this function requires the spleen. (iii) Vagus nerve stimulation significantly attenuates neutrophil surface CD11b levels only in the presence of an intact and innervated spleen. (iv) *α*7 nAChR^+^Gr1^+^ neutrophils are increased in the lungs and activation of *α*7 nAChR reduces neutrophil transalveolar migration in *E. coli* pneumonia. (v) Activation of *α*7 nAChR decreases proinflammatory cytokine production in neutrophils.	Reduction of neutrophils in the lung renders protective effects on sepsis and ALI	[5, 50, 83]

Mononuclear cells (MNCs)	Rats	POCD; metabolic syndrome	*α*7 nAChR agonists: PHA, *β*2 adrenergic agonist	(i) Under lipopolysaccharide LPS stimulation, TNF-*α* produced by splenic MNCs was 117% higher in LCR sham and 52% higher in LCR surgery compared with HCR sham and surgery rats. (ii) LPS-stimulated TNF-*α* production could not be inhibited by an *α*7 nAChR agonist LCR rat MNCs, whereas inhibition by the *β*2 adrenergic agonist, salmeterol, was significantly less (−35%) than that obtained in HCR rats.	Rats with the metabolic syndrome have ineffective CAP	[28]

Dendritic cells (DCs)	Mouse	Immature dendritic cells (imDCs); HBV immunotherapy	Nicotine	(i) Nicotine upregulated the expression of *α*7 nAChR by activating PI3K-Akt pathway in murine DCs. (ii) Nicotine stimulation could enhance DCs' ability of HBV-specific T cell proliferation and IL-12 secretion. (iii) Adoptive transfer of nicotine stimulated DCs could induce HBV-specific CTL priming *in vivo* and those CTL had cytolytic activities. (iv) Nicotine had equal efficiencies to 2 ng/mL IFN-*γ* in DCs-mediated T cell proliferation.	Beneficial effects for HBV immunotherapy	[107]

T lymphocytes; B lymphocytes	Mouse	Sepsis infection	*α*7 nAChR genetic depletion; bone marrow transplantation	(i) CD4^+^ T cell population that is stimulated by norepinephrine to release ACh. (ii) ChAT^+^ B cells release ACh after stimulation with sulfated cholecystokinin. (iii) *β*2-Adrenoreceptors of regulatory lymphocytes are essential for vagal neuromodulation of the innate immune system. (iv) Cholinergic lymphocytes reestablish splenic protection and the potential of cholinergic agonists to rescue immunocompromised animals from established sepsis. (v) Increased DSS-induced inflammation was associated with reduced CD4^+^CD25^+^Foxp3^+^ regulatory T cell numbers in recipients. Adoptive transfer of CD4^+^ T cells from vagotomized animals (but not CD4^+^ T cells from sham-operated controls) to naive DSS-treated recipients resulted in increased inflammatory scores.	T and B lymphocytes synthesize ACh, regulating neutrophil recruitment and innate immunity	[3, 77, 108–110]

Epithelial cells	Rats, human, and mouse	*In vitro* cell culture and lung injury models	*α*7 nAChR agonist: nicotine, genetic depletion of *α*7 nAChR	(i) Human type II alveolar epithelial cells express *α*7 nAChR. (ii) Nicotine activates and upregulates nicotinic acetylcholine receptors in bronchial epithelial cells. (iii) Human epithelial HEp-2 cells express *α*7 nAChR. Treatment of HEp-2 cells with nicotine after infection with bacteria resulted in a significant increase in *C. pneumoniae* inclusion numbers in cells. (iv) nAchR activation by topical agonist application or deletion of the nAChR antagonist catestatin reduced antimicrobial peptide (AMP) activity in skin extracts and increased susceptibility to methicillin-resistant *Staphylococcus aureus* and group A *Streptococcus *infections. (v) *α*7 nAChR is in fundamental cellular processes relevant to lung development, injury and repair, and carcinogenesis	In the lung epithelial cells, involvement of *α*7 nAChR in controlling bacteria growth, cell growth, and repair	[5, 78, 111–113]

Endothelial cells	Human Mouse	*In vitro* cell culture and sepsis model	*α*7 nAChR agonists: nicotine, CAP55	(i) HuMVECs express the cell surface *α*7 nAChR. (ii) ACh and nAChR agonists inhibit TNF-induced adhesion molecule expression by HuMVECs. (iii) ACh and nAChR agonists reduce TNF-induced chemokine production by endothelial cells. (iv) Changes in molecular (upregulation, affinity, and conformational states) and cellular (distribution, association with membranes) properties of the *α*7AChR related to angiogenesis (wound-repair cell migration) and atherogenesis (alterations in cholesterol content) were studied in living endothelial cells. (v) The nAChRs on endothelial cells modulate key angiogenic processes, including endothelial cell survival, proliferation, and migration.	Endothelial cell activation and leukocyte binding; angiogenesis; atherogenesis	[114–116]

Hematopoietic stem cells	Mouse	Sepsis	*α*7 nAChR Cre, Rosa26-Flox, YFP labeling; bone marrow transplantation	(i) In the adult, on average 20–25% of the total CD45^+^ myeloid and lymphoid cells of the bone marrow (BM), blood, spleen, lymph nodes, and Peyer's patches are *α*7 nAChR^+^lin^+^. (ii) This hematopoietic *α*7 nAChR^+^lin^+^ subpopulation is also found in Sca1^+^cKit^+^ BM cells. (iii) Both *α*7 nAChR^+^lin^+^ and *α*7 nAChR^+^lin^−^ BM cells can reconstitute the immune system of naïve irradiated recipient mice. (iv) Functionally the *α*7 nAChR^+^lin^+^ and *α*7 nAChR^+^lin^−^lineages differ in response to LPS challenge. (v) Production of IL-12/23(p40) was enhanced in the *α*7 nAChR^+^lin^+^ cells in response to LPS challenge.	*α*7 nAChR-expressing HSCs could repopulate during inflammation	[82]

Mesenchymal stem cells	Human	Cell culture	*α*7 nAChR agonists: nicotine	(i) MSCs also expressed *α*7 nAChR. (ii) Stimulation of MSCs with the nicotinic receptor agonist nicotine and the muscarinic receptor agonist muscarine induced immediate and transient increases in intracellular Ca^2+^ concentration. (iii) At nontoxic concentrations, nicotine increased spontaneous migration of hMSCs, whereas chemotaxis of hMSCs toward C3a and bFGF *in vitro* and migration of intravenously infusion hMSCs into bone marrow and spleen *in vivo* were inhibited. (iv) The antagonist for the alpha 7 homopolymer, bungarotoxin, blocked the inhibitory effect of nicotine on chemotactic factor-induced migration of hMSCs.	Regulation of MSC migration	[117, 118]

Endothelial progenitor cells	Human Mouse	Cell culture and ischemia model	*α*7 nAChR agonists: nicotine	(i) EPCs expressed *α*7 nAChR. (ii) Incubation with nicotine enhanced viable, migratory, adhesive, and *in vitro* vasculogenesis capacity of late EPCs. (iii) The effect of nicotine on late EPCs can be attenuated by mecamylamine or *α*-bungarotoxin. (iv) Nicotine treatment increased the number of EPCs in the bone marrow and spleen and increased their incorporation into the vasculature of ischemic tissue. Administration of nicotine increased markers of EPC mobilization.	Mobilization of EPCs facilitates angiogenesis	[119, 120]

Fibroblast	Human Mouse	Arthritis patients and models	Immunofluorescence; depletion of *α*7 nAChR	(i) Fibroblasts from synovial tissue of arthritis patients expressed *α*7 nAChR. (ii) In *α*7 nAChR knockout mice, a significant increase in the incidence and severity of arthritis and increased synovial inflammation and joint destruction were seen.	Activation of *α*7 nAChR is protective in arthritis	[121, 122]

HMGB1: high-mobility group box 1 protein; RAGE: advanced glycation end products; GTS-21: 3-(2,4-dimethoxybenzylidene)-anabaseine dihydrochloride; EPCs: endothelial progenitor cells; MSCs: mesenchymal stem cells; HSCs: hematopoietic stem cells; POCD: postoperative cognitive decline; CHAT: choline acetyltransferase; DSS: dextran sulfate sodium; HCR: high capacity runners; LCRs: low capacity runners.

**Table 3:** Modulatory effects of activation of *α*7 nAChR on signaling pathways.

Pathways	Species	Models	Interventions	Major results	Outcome	References
TLRs MyD88	Human Rats	Cell culture: epithelial cells; macrophages; or monocytes	TLRs agonists; *α*7 nAChR agonists: nicotine; or GTS-21 pretreatment	(i) In human monocytes, GTS-21 attenuated TLR2, TLR3, TLR4, TLR9, and RAGE-mediated TNF production. (ii) GTS-21 decreased TNF levels in endotoxin-stimulated whole blood obtained from patients with severe sepsis. (iii) GTS-21 downregulated monocyte cell-surface expression of TLR2, TLR4, and CD14. (iv) An anti-inflammatory effect of nicotine on splenocytes isolated from control Wistar Kyoto rats (inhibition of interleukin-6 release) is reversed to a proinflammatory increase of interleukin-6 release from splenocytes of young, prehypertensive, and spontaneously hypertensive rats (SHRs). (v) The serum levels of both IL-6 and IL-1*β* in response to TLR7/8 activation with Clo97 (intraperitoneally) were markedly suppressed by the subcutaneous infusion of nicotine in WKY rats and conversely significantly enhanced in SHRs.	Downregulation of MyD88 and TLR signaling	[66, 94, 123]

NF-*κ*B	Mouse	Sepsis; cell culture of macrophages; RAW cells	Nicotine; choline	(i) In bronchial epithelial cells, nicotine decreased MyD88 protein, NF-*κ*B p65 protein, NF-*κ*B activity, and p-I-*κ*B*α* expression induced by CE or LPS. (ii) GTS-21 inhibits NF-*κ*B activation in endotoxin-stimulated RAW cells in a dose-dependent manner. (iii) Nicotine inhibits p65 NF-*κ*B nuclear translocation in the BAL proinflammatory cells in acid-induced ALI mouse model. (iv) Inhibition of STAT3 phosphorylation mimicked the *α*7 nAChR signaling, inhibiting NF-*κ*B and cytokine production in macrophages. (v) Choline dose dependently suppressed NF-*κ*B activation in response to endotoxin.	Suppress activation of NF-*κ*B	[5, 97, 124, 125]

*Jak-Stat3 *	Mouse; human	Macrophages; endothelial cells; cell culture; sepsis; peritonitis	GTS-21; nicotine; depletion; or blockade of *α*7 nAChR	(i) STAT3 was phosphorylated by the tyrosine kinase Jak2 that was recruited to the *α*7 nAChR. (ii) The anti-inflammatory effect of nicotine required the ability of phosphorylated STAT3 to bind and transactivate its DNA response elements. (iii) In a mouse model of intestinal manipulation, stimulation of the vagus nerve ameliorated surgery-induced inflammation and postoperative ileus by activating STAT3 in intestinal macrophages. (iv) GTS-21 inhibits proinflammatory cytokine release independent of the Toll-like receptor stimulated via a transcriptional mechanism involving JAK2 activation. The inhibitor *α*-BTX could reverse these effects. (v) Both *α*7 nAChR activation and inhibition of JAK2 blunt STAT3 phosphorylation. (vi) Inhibition of STAT3 protein expression enhanced cytokine production and abrogated *α*7 nAChR signaling. (vii) *α*7 nAChR controls TNF production in macrophages through a mechanism that requires STAT3 protein expression but not its tyrosine phosphorylation. (viii) *In vivo*, inhibition of STAT3 tyrosine phosphorylation by stattic prevented systemic inflammation and improved survival in experimental sepsis. (ix) Cholinergic agonists suppress IL-6-mediated endothelial cell activation through the JAK2/STAT3 pathway. (x) Nicotine and GTS-21 treatment decreased levels of SOCS3 in activated endothelial cells. (xi) MicroRNA-124 mediates the cholinergic anti-inflammatory action through Stat3 and inhibits the production of proinflammatory cytokines.	Activation of *α*7 nAChR is protective through Jak2-STAT3 signaling pathway in macrophages and endothelial cells	[14, 94, 95, 97, 126]

PI3k-AKT1	Rats Mouse	Cell culture: neurons; macrophages; monocytes; CLP sepsis	Nicotine or DMPP; MLA; PI3K inhibitors treatment	(i) Nicotine improved sepsis-induced mortality, attenuated organ failure, and suppressed inflammatory cytokines, which were abolished by MLA. (ii) In macrophages, nicotine enhanced PI3K/AKT1 activation and reduced PU.1 activity and TLR4 expression. MLA and PI3K inhibitors blocked this effect of nicotine. (iii) In brain cortex primary cells, the *α*7 nAChR was physically associated with the PI3K p85 subunit and Fyn. Activation of *α*7 nAChR phosphorylates AKT1. (iv) Nicotine stimulation of *α*7 nAChR transduces signals to PI3K and AKT1 via JAK2 in a cascade. (v) Coimmunoprecipitation of PI3K and nAChR *α*3, −4, and −7 subunits. Treatment of monocytes for 24 h with 10, 20, and 40 *μ*M DMPP dose dependently inhibited TNF release.	Neuroprotective effect and anti-inflammation	[127–130]

P38MAPK	Rats Human	PC12 cells, rat pheochromocytoma cells; SH-SY5Y neuroblastoma cells	Nicotine, choline, GTS-21, SSR-180711A, and PNU-282987	(i) *α*7 nAChR agonist-triggered Ca^2+^ transient in PC12 cells induces activation of CaMKII, leading to sequential phosphorylation of p38 MAPK, MEK1/2, ERK1/2, and CREB. (ii) TLR and DR signaling, such as PI3K/AKT signaling, p38 signaling, and ERK signaling, were also significantly modulated by nicotine.	Cognition	[131, 132]

ERK1/2	Rats	PC12 cells	PNU-282987, PNU-120596	(i) Robust induction of ERK phosphorylation followed exposure of PC12 cells to the selective agonist PNU-282987 in the presence of the alpha 7 nAChR modulator PNU-120596. (ii) ERK phosphorylation was transient and was attenuated by the selective antagonist MLA. (iii) Consistent with allosteric modulation of alpha 7 nAChRs, PNU-120596 enhanced both the agonist potency and efficacy in activating ERK.	Cognition	[133]

DMPP: dimethylphenylpiperazinium; CE: cigarettes extract; JAK2: Janus kinase 2; PI3K: phosphatidylinositol 3-kinase; WKY: Wistar Kyoto; SHRs: spontaneously hypertensive rats; DR: death receptor; MLA: methyllycaconitine.
